# Adjunctive Use of Cenobamate in Paediatric Drug-Resistant Epilepsy: A Real-World, Single-Centre Experience

**DOI:** 10.3390/jcm15031218

**Published:** 2026-02-04

**Authors:** Barbara Oleksy, Agata Lipiec, Alicja Goszczańska-Ciuchta, Joanna Żebrowska, Magdalena Bosak, Aleksandra Kuźniar-Pałka, Hanna Mazurkiewicz, Elżbieta Lipińska, Tomasz Mazurczak, Elżbieta Stawicka

**Affiliations:** 1 Clinic of Paediatric Neurology, Institute of Mother and Child, Kasprzaka 17A, 01-211 Warsaw, Poland; agata.lipiec@imid.med.pl (A.L.); alicja.goszczanska@imid.med.pl (A.G.-C.); joanna.zebrowska@imid.med.pl (J.Ż.); aleksandra.kuzniar@imid.med.pl (A.K.-P.); hanna.mazurkiewicz@imid.med.pl (H.M.); elzbieta.lipinska@imid.med.pl (E.L.); tomasz.mazurczak@imid.med.pl (T.M.); elzbieta.stawicka@imid.med.pl (E.S.); 2 Department of Neurology, Medical College, Jagiellonian University, 31-007 Cracow, Poland; magdalena.bosak@uj.edu.pl

**Keywords:** cenobamate, paediatric population, real-world data, drug-resistant epilepsy

## Abstract

**Background/Objectives:** Epilepsy represents one of the most common chronic neurological disorders in children, with a considerable proportion of patients exhibiting resistance to pharmacotherapy despite the advent of novel antiseizure medications (ASMs) in recent decades. This retrospective study assesses the off-label administration of cenobamate—a newly approved antiseizure medication (ASM) for focal seizures in adults—in a cohort of paediatric patients with drug-resistant epilepsy at a single neurology centre. **Methods:** Clinical outcomes were reviewed retrospectively for 18 children who received cenobamate for at least 6 months. **Results:** Eighteen paediatric patients with drug-resistant epilepsy received cenobamate therapy at a neurology centre. The mean age was 164.6 months, and each patient had previously trialled an average of 8.7 antiseizure medications. During a follow-up period of up to 29 months, 39% of participants achieved complete seizure freedom, while five additional patients experienced a seizure reduction exceeding 80%. Concomitant clobazam use was common among the cohort. Adverse events were reported in 78% of patients, predominantly somnolence, though these were generally transient or manageable. One patient developed a temporary exacerbation of seizures, which resolved following a dosage adjustment. Many patients were able to reduce or discontinue other ASMs during the observation period. **Conclusions:** Cenobamate demonstrated acceptable tolerability in this paediatric cohort, and seizure improvements were observed in a subset of patients. Further clinical trials are warranted to comprehensively evaluate the efficacy and safety profile of cenobamate in this patient population.

## 1. Introduction

Epilepsy is among the most prevalent chronic neurological disorders, affecting approximately 0.5–1% of the paediatric population, with the highest incidence observed during the first year of life [1].

Early-onset epilepsy is associated with an increased risk of drug resistance, defined as the failure to achieve seizure freedom despite adequate trials of at least two appropriately selected and dosed antiseizure medications (ASMs) [2].

For selected patients who meet specific criteria, epilepsy surgery and the ketogenic diet constitute viable treatment alternatives [3,4]. However, most patients require long-term pharmacological management.

Despite the development of new ASMs over recent decades, the rate of drug resistance has remained largely unchanged [5], continuing to pose a major therapeutic challenge, particularly in paediatric populations.

Cenobamate (CNB) is a novel antiseizure medication approved by the U.S. Food and Drug Administration (FDA) in 2019 for the treatment of focal seizures in adults, and by the European Medicines Agency (EMA) in 2021 for the treatment of focal seizures with or without secondary generalisation in adults with drug-resistant epilepsy (DRE). Cenobamate exerts a dual mechanism of action: it inhibits persistent voltage-gated sodium currents and acts as a positive allosteric modulator of GABA_A_ receptors [6].

Clinical trials [7] and retrospective studies [8] have demonstrated cenobamate’s promising efficacy and tolerability in adults. Although CNB has not yet been approved for use in the paediatric population, limited real-world retrospective data suggest successful off-label administration in children, indicating its potential applicability in this group [9,10,11].

The present study summarises the experience with off-label cenobamate treatment in 18 children with DRE managed at a single paediatric neurology centre, presenting descriptive findings on clinical outcomes, tolerability and practical insights from real-world clinical use.

## 2. Materials and Methods

A retrospective analysis was conducted involving all patients under 18 years of age with DRE who received cenobamate treatment for at least 6 months at a single paediatric neurology centre.

Clinical data were extracted from medical records and included patient demographics (age, sex), duration of epilepsy, ASMs previously used, concomitant ASMs during cenobamate therapy, cenobamate dosage, treatment duration, and seizure type (focal [FS], focal to bilateral tonic–clonic [FBTC], or generalised tonic–clonic [GTC]).

Changes in seizure frequency and adverse events were assessed based on patient and caregiver reports. Seizure frequency was determined by calculating the weighted average of seizure counts during each of the three months prior to initiating cenobamate therapy. This baseline was then compared with the weighted average for the last three-month period of observation, with results expressed as percentage change.

The adverse events (AEs) were assessed for severity, causality, and seriousness. Severity was graded according to Common Terminology Criteria for Adverse Events (CTCAE), Version 5.0 [12], causality was assessed using the Naranjo algorithm [13], and seriousness was determined based on international pharmacovigilance criteria (International Council for Harmonisation E2A [ICH E2A]) [14].

Descriptive statistics were used for data analysis.

All data were anonymized prior to analysis. The study was approved by the institutional ethics committee. Due to the retrospective nature of the study, the requirement for informed consent was waived.

## 3. Results

Eighteen patients (10 females, 8 males) aged 84–215 months at treatment onset (mean 164.6 months; median [IQR] 179.5 [132–202]) were included. The mean age at seizure onset was 46.7 months (range 4–130 months; median [IQR] 41 [18–72]), with a mean interval of 117.8 months (range 26–178 months; median [IQR] 120 [87–152]) between seizure onset and cenobamate initiation. All patients had drug-resistant epilepsy of diverse aetiologies, including cortical malformations, genetic syndromes, autoimmune conditions, and unknown causes. Prior to cenobamate therapy, patients had received a mean of 8.7 ASMs (range 4–16; median [IQR] 9 [6–10]). On average, patients experienced 88 focal seizures per month (range 0–455; median [IQR] 56 [3.5–100]) and 9.6 FBTC or GTC seizures (range 0–140; median [IQR] 0 [0–4]). Clobazam, lamotrigine, and valproic acid were the ASMs most frequently used concurrently (see Table 1).

Cenobamate was titrated according to the manufacturer’s recommendations and administered once daily. The initial dose was 12.5 mg; in four children weighing < 40 kg, a slower titration schedule was implemented, with doses adjusted to body weight (6.25 mg → 12.5 mg → 25 mg → 50 mg → 75 mg → 100 mg → 150 mg). The mean final dose was 184 mg (range 100–300 mg; median [IQR] 175 [150–218.75]), corresponding to 3.6 mg/kg (range 2.1–6.81 mg/kg; median [IQR] 3.37 [2.85–4.25]).

### 3.1. Efficacy

The mean follow-up duration was 13 months (range 6–29 months; median [IQR] 12, [8–17]), with 9 patients treated for ≥13 months. Two patients achieved >90% seizure reduction, three patients experienced ≥80% reduction, and seven patients (39%) were seizure-free at the last observation. None continued to experience GTC or FBTC seizures during cenobamate treatment.

Fifteen patients (83%) received concomitant clobazam during CNB therapy. Nine were already taking clobazam at baseline, in four of these, the dose was reduced during treatment and one patient discontinued CLB. In six additional patients, clobazam was introduced later. Among the seven seizure-free patients, six were receiving both CNB and clobazam. In the three patients who did not receive clobazam during the study period, one patient was seizure-free, while the remaining two showed an unsatisfactory seizure response, with no seizure reduction and a 25% seizure reduction, respectively.

Most patients (89%) had previously been treated with six or more ASMs. In 10 patients (56%), dose reduction of concomitant ASMs was possible, and 12 patients (67%) discontinued at least one ASM during follow-up.

### 3.2. Adverse Events

Adverse events (AEs) occurred in 14 patients (78%). AEs were graded according to CTCAE v5.0 and assessed for causality and seriousness as described in Section 2. All AEs except for suicidal ideation were CTCAE Grade 1–2, non-serious, and the causality of cenobamate was considered possible. The most frequently reported AE was somnolence (n = 12), primarily observed during titration, it was usually transient or resolved after shifting CNB administration to evening dosing. Suicidal ideation was classified as a serious adverse event due to its clinical significance, independent of CTCAE grade 1. No other serious adverse events were observed, and no AE led to treatment discontinuation.

Notably, none of the three patients who were not receiving clobazam reported any adverse events.

Patient 9, receiving lamotrigine and clobazam, experienced tic disorder and suicidal ideation at a cenobamate dose of 150 mg (3.33 mg/kg). These symptoms resolved after reducing the CNB dose to 100 mg. Following discontinuation of LTG and CLB initiated by the caregivers, and subsequent re-titration of CNB to 125 mg, the patient has remained seizure-free at the most recent follow-up (one month after reaching 125 mg).

No severe dermal reactions were detected.

A transient seizure exacerbation occurred in patient 3 at a dose of 100 mg (1.54 mg/kg), but increasing the dose to 150 mg CNB led to seizure reduction and, ultimately, seizure freedom after substitution of clonazepam with clobazam.

In one patient with Dravet syndrome, cenobamate demonstrated limited long-term efficacy. Attempts to discontinue CNB resulted in seizure worsening, and treatment was continued at 150 mg until fenfluramine was introduced, after which CNB was gradually tapered.

## 4. Discussion

This study presents real-world data on cenobamate use in paediatric patients with drug-resistant epilepsy of heterogeneous aetiologies. In this descriptive cohort, seizure freedom was observed in 39% of patients and a ≥50% seizure reduction in an additional eight patients during cenobamate treatment, with overall tolerability considered acceptable. Notably, all patients experienced cessation of GTC or FBTC seizures during therapy.

These findings are consistent with previously published data demonstrating cenobamate’s beneficial impact on seizure burden in children. In our cohort, seizure freedom was achieved in 39% of patients, a higher proportion compared with prior reports (21.1–31.3%) [9,10]. This discrepancy may be attributed to the small sample size and short follow-up period in some cases, as well as the fact that seizure frequency was assessed using patient and caregiver reports, which may limit the precision of efficacy estimates. Another key consideration is the frequent use of clobazam as part of heterogeneous polytherapy regimens alongside cenobamate, which may have contributed to seizure outcomes and adverse events.

CNB has a complex interaction profile due to its effects on hepatic drug-metabolising enzymes. CNB inhibits CYP2C19, which can markedly increase plasma concentrations of phenytoin, phenobarbital and N-desmethylclobazam (the active metabolite of clobazam), often leading to enhanced CNS effects such as somnolence and fatigue [15,16]. Clinically, this may necessitate dose adjustments of clobazam to maintain tolerability. In our cohort all patients reporting somnolence (n = 12) were on cenobamate-clobazam combination therapy. Of note is also the fact that patients not taking clobazam concomitantly (n = 3) did not report any AEs.

On the other hand, the concomitant use of clobazam in most patients may have contributed to improved outcomes, as prior studies have shown synergistic effects between CNB and clobazam. It is suspected that, beyond the pharmacokinetic interaction, there is a synergistic pharmacodynamic interaction between clobazam and cenobamate, contributing to superior anticonvulsant effect [16]. Of note, six of seven seizure-free patients in our cohort were receiving concomitant CNB and clobazam.

Among patients not receiving clobazam during the study period, one achieved seizure freedom, while the remaining two showed unsatisfactory seizure reduction. One of these patients was diagnosed with Dravet syndrome. Similar observations have been reported previously, suggesting that cenobamate may have limited effects in this epilepsy subtype [17].

CNB also induces CYP3A4 and glucuronidation enzymes, which can reduce plasma levels of other ASMs such as carbamazepine or lamotrigine, requiring monitoring and potential dose modification [15].

Pharmacodynamic interactions between cenobamate and other sodium channel–blocking antiseizure medications may increase the risk of central nervous system AEs without clinically relevant changes in plasma drug concentrations [15].

These pharmacokinetic and pharmacodynamic interactions are important considerations when implementing CNB in polytherapy for DRE, particularly regarding sedation risk in patients receiving concomitant CLB.

Transient seizure exacerbation during the titration phase, observed in one patient, was consistent with previously reported experiences [9].

Neuropsychiatric adverse events, such as the tic disorder and suicidal ideation observed in patient 9, are clinically relevant, particularly in the context of polytherapy with lamotrigine and clobazam. Although the exact causative agent is uncertain, cenobamate may have contributed, but the potential influence of concomitant medications and drug–drug interactions cannot be excluded. According to the Naranjo algorithm, all three medications were assessed as possible causes. Soto-Insuga et al. reported two cases of suicidal ideation that resulted in discontinuation of cenobamate [10].

These findings highlight the importance of careful monitoring for neuropsychiatric adverse events, especially when multiple antiseizure medications are administered concurrently. Overall, the safety profile observed in our cohort aligns with previously published data, with mostly mild to moderate events [10,11]. Importantly, neuropsychiatric symptoms, such as suicidal ideation, require vigilance and thorough clinical discussion.

In our cohort, adjustments to polytherapy, including dose reduction or discontinuation of other ASMs, occurred in all but two patients after cenobamate initiation.

In summary, while our findings suggest that cenobamate may be an effective and well-tolerated therapeutic option for children with drug-resistant epilepsy, it is important to interpret these results with caution. The limited sample size and heterogeneity of the cohort restrict the strength of the conclusions that can be drawn. Further research involving larger cohorts is needed to better characterise the clinical profile of cenobamate, while prospective controlled clinical trials are required to establish cenobamate’s safety, efficacy, and optimal dosing strategies in the paediatric population.

## Figures and Tables

**Table 1:** Patients treated with Cenobamate.

No.	Sex (M, F)	Age at Epilepsy Onset (Months)	Age at CNB Introduction (Months)	Epilepsy Duration (Months)	Intellectual Disability	Epilepsy Aetiology	Seizure Type		Prior ASM (n)	Non-Pharmacological Treatment	ASM Used Together with CNB (mg)	Clobazam Changes (mg) (+ Adding − Reduction 0 No Change X Without CLB)	CNB End Dose (mg)	CNB Dose in mg/kg	Follow Up in Months	Seizure Reduction	Discontinued ASM	ASM Reduction (Current Dose)	Adverse Events
							GTCS, FBTC	FS											
1	F	4	84	80	moderate	FCD		✓	9	VNS	LTG 150, NZP 5	+ CLB 12.5	150	6.81	21	92%	NZP	none	somnolence, sialorrhoea, gait disturbance
2	F	51	179	128	none	unknown		✓	10	KD	LTG 300, VPA 300, LCM 175	X	200	3.7	16	0%	LCM, VPA	none	none
3	M	7	180	173	profound	EFTUD2		✓	8		VPA 1100, CZP 1.5, BRI 200, PB 150, LTG 100	+ CLB 30	200	3.1	8	100%	PB, LTG, CZP	none	none
4	M	130	156	26	none	unknown		✓	6		VGB 1000, VPA 850, LCM 350, CLB 5	0	150	3.33	8	100%	VGB	LCM 175	Somnolence, disturbance in attention
5	F	31	132	101	moderate	CHD2		✓	6		CBZ 600, CLB 10	− CLB 5	100	2.85	7	100%	CBZ	CLB 5	alopecia
6	M	24	202	178	severe	unknown		✓	8		LTG 375, CZP 0.5, CLB 10	0	250	2.58	7	100%	CZP	LTG 300	somnolence
7	F	56	212	156	none	RE		✓	10	VNS, S, KD	LTG 400, CLB 20	0	300	3.4	11	97%	none	none	somnolence
8	M	72	186	114	moderate	perinatal		✓	9		VPA 1000, LCM 300, TPM 200, CLB 20	− CLB 10	100	2.1	10	85%	none	LCM 200, CLB 10	somnolence
9	F	24	104	80	none	unknown		✓	6		LTG 250, CLB 5	− CLB 5	125	2.78	16	70%	LTG, CLB	none	Suicidal ideation, tic disorder
10	F	74	164	90	moderate	unknown		✓	9		VPA 700, FFA 17.6, CLB 15	− CLB 2.5	150	3.57	8	50%	none	CLB 12.5	somnolence
11	M	7	154	147	moderate	DS	✓	✓	12	VNS	VPA 1000, LEV 2500, FFA 12	X	150	2.67	20	25% **	none	LEV 1500	none
12	F	18	105	87	severe	CFC	✓	✓	16		STM 200, VPA 600	+ CLB 7.5	112.5	4.33	29	83%	STM	none	somnolence
13	F	24	196	172	none	unknown	✓	✓	4		LEV 2000, OXC 1200	X	200	2.94	7	100%	OXC	none	none
14	F	124	202	78	none	RE	✓	✓	5		LTG 300, BRI 175	+ CLB 5	300	4.61	13	67%	BRI	LTG 200	somnolence
15	M	60	212	152	severe	KMT5B	✓	✓	7		LTG 400, CLB 10	− CLB 5	250	4.25	15	100%	none	LTG 200, CLB 5	somnolence
16	M	4	94	90	profound	SCN8A	✓	✓	10		CBZ 900, ZNS 200, CLB 17.5	0	150	5	6	89% *	none	none	somnolence
17	F	59	185	126	none	unknown	✓	✓	11		VPA 1200, LTG 200, LEV 2000, CZP 1	+ CLB 10	200	3.8	17	100%	CZP	VPA 500	somnolence
18	M	72	215	143	severe	unknown	✓	✓	10		AZA 500, LTG 175, VPA 1200, CZP 0.5	+ CLB 10	225	3.04	17	49%	AZA, CZP	LTG 150	somnolence, decreased appetite

Abbreviations: ASM, antiseizure medication; AZA, acetazolamide; BRI, brivaracetam; CBD, cannabidiol; CBZ, carbamazepine; CFC, cardio-facio-cutaneous syndrome; CLB, clobazam; CNB, cenobamate; CZP, clonazepam; DS, Dravet syndrome; F, female; FBTC, focal to bilateral tonic–clonic seizures; FCD, focal cortical dysplasia; FFA, fenfluramine; FS, focal seizures; GTCS, generalised tonic–clonic seizures; KD, ketogenic diet; LCM, lacosamide; LEV, levetiracetam; LTG, lamotrigine; M, male; NZP, nitrazepam; OXC, oxcarbazepine; PB, phenobarbitone; RE, Rasmussen’s encephalitis; S, epilepsy surgery; STM, sultiam; TPM, topiramate; VGB, vigabatrin; VNS, vagus nerve stimulation; VPA, valproic acid; ZNS, zonisamide; ✓, presence of the specified seizure type. * countable motor seizures. ** CNB discontinued due to lack of efficacy.

## Data Availability

The original contributions presented in this study are included in the article. Further inquiries can be directed to the corresponding author.

## Abbreviations

The following abbreviations are used in this manuscript: AEs, adverse events; ASM, antiseizure medication; AZA, acetazolamide; BRI, brivaracetam; CBD, cannabidiol; CBZ, carbamazepine; CFC, cardio-facio-cutaneous syndrome; CLB, clobazam; CNB, cenobamate; CTCAE, Common Terminology Criteria for Adverse Events; CZP, clonazepam; DRE, drug-resistant epilepsy; DS, Dravet syndrome; EMA, European Medicines Agency; F, female; FBTC, focal to bilateral tonic–clonic seizures; FCD, focal cortical dysplasia; FFA, fenfluramine; FDA, Food and Drug Administration; FS, focal seizures; GTCS, generalised tonic–clonic seizures; ICH E2A, International Council for Harmonisation E2A; KD, ketogenic diet; LCM, lacosamide; LEV, levetiracetam; LTG, lamotrigine; M, male; NZP, nitrazepam; OXC, oxcarbazepine; PB, phenobarbitone; RE, Rasmussen’s encephalitis; S, epilepsy surgery; STM, sultiam; TPM, topiramate; VGB, vigabatrin; VNS, vagus nerve stimulation; VPA, valproic acid; ZNS, zonisamide.

## References

[1] Aaberg K.M., Gunnes N., Bakken I.J., Søraas C.L., Berntsen A., Magnus P., Lossius M.I., Stoltenberg C., Chin R., Surén P. (2017). Incidence and Prevalence of Childhood Epilepsy: A Nationwide Cohort Study. Pediatrics.

[2] Kwan P., Arzimanoglou A., Berg A.T., Brodie M.J., Allen Hauser W., Mathern G., Moshé S.L., Perucca E., Wiebe S., French J. (2010). Definition of drug resistant epilepsy: Consensus proposal by the ad hoc Task Force of the ILAE Commission on Therapeutic Strategies. Epilepsia.

[3] Jehi L., Jette N., Kwon C., Josephson C.B., Burneo J.G., Cendes F., Sperling M.R., Baxendale S., Busch R.M., Triki C.C. (2022). Timing of referral to evaluate for epilepsy surgery: Expert Consensus Recommendations from the Surgical Therapies Commission of the International League Against Epilepsy. Epilepsia.

[4] Verrotti A., Iapadre G., Di Francesco L., Zagaroli L., Farello G. (2020). Diet in the treatment of epilepsy: What we know so far. Nutrients.

[5] Löscher W., Klein P. (2021). The Pharmacology and Clinical Efficacy of Antiseizure Medications: From Bromide Salts to Cenobamate and Beyond. CNS Drugs.

[6] Roberti R., De Caro C., Iannone L.F., Zaccara G., Lattanzi S., Russo E. (2021). Pharmacology of Cenobamate: Mechanism of Action, Pharmacokinetics, Drug–Drug Interactions and Tolerability. CNS Drugs.

[7] Krauss G.L., Klein P., Brandt C., Lee S.K., Milanov I., Milovanovic M., Steinhoff B.J., Kamin M. (2020). Safety and efficacy of adjunctive cenobamate (YKP3089) in patients with uncontrolled focal seizures: A multicentre, double-blind, randomised, placebo-controlled, dose-response trial. Lancet Neurol..

[8] Bosak M., Podraza H., Włoch-Kopeć D., Rysz A., Wężyk K., Grabska-Radzikowska K., Sobolewski P., Siwek T., Kurkowska-Jastrzębska I., Służewska-Niedźwiedź M. (2025). Efficacy and safety of Cenobamate: A multicenter, retrospective evaluation of real-world clinical practice. Seizure Eur. J. Epilepsy.

[9] Makridis K.L., Bast T., Prager C., Kovacevic-Preradovic T., Bittigau P., Mayer T., Breuer E., Kaindl A.M. (2022). Real-World Experience Treating Pediatric Epilepsy Patients With Cenobamate. Front. Neurol..

[10] Soto-Insuga V., Carbó A.V., Pinzón-Acevedo A.G., González-Alguacil E., Peñas J.J.G., Jamardo A.S., Aznar-Laín G., Ibáñez-Micó S., Martínez H.A., Buenache R. (2025). Real-world use of cenobamate in pediatric focal epilepsies and developmental epileptic encephalopathies: A multicenter retrospective series. Epilepsia.

[11] Varughese R.T., Shah Y.D., Karkare S., Kothare S.V. (2022). Adjunctive use of cenobamate for pediatric refractory focal-onset epilepsy: A single-center retrospective study. Epilepsy Behav..

[12] National Institutes of Health, National Cancer Institute (2017). Common Terminology Criteria for Adverse Events (CTCAE) v5.0. https://dctd.cancer.gov/research/ctep-trials/for-sites/adverse-events.

[13] Naranjo C.A., Busto U., Sellers E.M., Sandor P., Ruiz I., Roberts E.A., Janecek E., Domecq C., Greenblatt D.J. (1981). A method for estimating the probability of adverse drug reactions. Clin. Pharmacol. Ther..

[14] International Council for Harmonisation of Technical Requirements for Pharmaceuticals for Human Use (ICH) (1994). Clinical Safety Data Management: Definitions and Standards for Expedited Reporting (E2A).

[15] Perucca E., Bialer M., White H.S. (2023). New GABA-Targeting Therapies for the Treatment of Seizures and Epilepsy: I. Role of GABA as a Modulator of Seizure Activity and Recently Approved Medications Acting on the GABA System. CNS Drugs.

[16] Osborn M., Abou-Khalil B. (2023). The cenobamate-clobazam interaction- evidence of synergy in addition to pharmacokinetic interaction. Epilepsy Behav..

[17] Cagigal R., Romero-Del-Rincon C., Fernandez-Perrone A., Cruz R., Møller R.S., Aledo-Serrano A. (2025). Lack of effectiveness and seizure worsening with cenobamate in pediatric patients with Dravet syndrome. Epilepsia.

